# A Scoping Review of Amenable Patient-Specific Predictors of Treatment Failure in the Treatment of Anxiety and Depressive Disorders

**DOI:** 10.32872/cpe.17335

**Published:** 2026-05-29

**Authors:** Vivian Peerbooms, Th. Michael van den Boogaard, Matthias J. Wieser, Colin van der Heiden

**Affiliations:** 1 Department of Anxiety Disorders, Outpatient Treatment Center PsyQ, Rotterdam, The Netherlands; 2 Department of Psychology, Education and Child Studies, Erasmus University Rotterdam, Rotterdam, The Netherlands; 3 Department of Affective Disorders, Outpatient Treatment Center PsyQ, The Hague, The Netherlands; Friedrich-Alexander-Universität Erlangen-Nürnberg, Erlangen, Germany

**Keywords:** treatment failure, predictors, scoping review, anxiety disorders, depressive disorders

## Abstract

**Background:**

By identifying predictors of treatment failure that are susceptible to change (amenable), we can move towards studying ways to decrease the odds of treatment failure, e.g., by targeting these predictors before treatment, adapting interventions, accordingly, choosing more suitable treatments, or preparing patients better for psychotherapy. While treatment success, within anxiety and depressive disorders, has been studied extensively, it seems that treatment failure is overlooked, even while we know that about one third of the treatment population shows no benefit in treatment.

**Method:**

In order to review the available body of knowledge concerning amenable patient-specific predictors for treatment failure, we conducted a literature search in PubMed, PsycInfo, Embase, and Medline, following the Prisma-ScR guidelines. Thirty articles met the inclusion criteria and are summarized in this review. Conclusions were drawn for scientific and clinical implications.

**Results:**

Predictors of treatment failure that are replicated or are significant in multiple studies are low treatment expectancy, high neuroticism, low use of social support, low outcome expectancy, and low perceived social support. Treatment failure is hard to define, and very few studies are replicated. There are predictors that are studied in multiple articles, but they are measured with different instruments, or in very small or specific patient samples, therefore it is difficult to compare findings from different studies.

**Conclusions:**

There are no predictors that stand out as overall strong amenable predictors of treatment failure. Possible predictors are high neuroticism, low treatment expectancies, and low use of social support. Future research should focus on replicating studies to confirm these predictors of treatment failure.

## Highlights

- Reducing treatment failure might improve treatment quality and shorten duration.
- Possible predictors are high neuroticism, low treatment expectancies, and low use of social support.
- There are no predictors that stand out as overall strong amenable predictors of treatment failure.
- Replication studies are necessary to identify predictors of treatment failure.

With a worldwide prevalence of 3.8% and 4%, anxiety and depressive disorders are the most common mental health disorders (World Health Organization [WHO], 2023a, 2023b). Despite the availability of effective treatments, treatment failure remains a major problem. Average response rates are still unsatisfactory, with 48% response for patients with major depressive disorder (Cuijpers et al., 2014) and 50% response of CBT in anxiety disorders (Loerinc et al., 2015). These figures emphasize the need to identify factors that predict the (in)effectiveness of treatment. Understanding these predictors can help reduce treatment failure by adapting interventions, selecting more suitable treatments, or better preparing patients for psychotherapy.

Remarkably, despite the abundance of studies reporting on predictors of treatment success (protective factors), relatively few studies report on treatment failure (risk factors; Dandachi-FitzGerald et al., 2023). However, treatment failure is often not well operationalized or clearly defined and tends to be used as an umbrella term for unwanted outcomes such as drop-out, non-response, attrition, deterioration, or poor treatment outcome (Oasi & Werbart, 2020). This lack of consensus complicates research into this subject. Similarly, the term response rates, which is often used to describe clinical effectiveness, lacks a consistent definition and calculation method across studies.

So far, studies into predictors of poor treatment outcome or non-response mainly confine themselves to demographic or disorder-specific features (e.g., duration or severity of symptoms) associated with treatment failure (Edmonds et al., 2018; McDevitt-Petrovic et al., 2020). Most of these predictors are the focus of the therapy itself and not changeable with other interventions (e.g., decreasing severity of symptoms is one important goal of therapy). However, amenable predictors may be more useful in clinical practice, as they can be influenced before therapy starts to prevent treatment failure. Eilertsen and Eilertsen (2023) suggest that focusing on changeable predictors is preferable, also for ethical reasons, because such knowledge can help to adapt treatments proactively. For example, van den Boogaard (2012) showed that drop-out rates in interpersonal therapy for depression decreased when patient-treatment compatibility was improved through a short intervention before therapy. This may as well be the case for more predictors like motivation, treatment attitude, or expectancies about treatment.

Although little is known regarding the mechanism of change in psychotherapy, a key to the black box of psychotherapy might be the distinction of trait-like and state-like components of the mechanisms of change (Zilcha-Mano, 2021). Mechanisms of change, or process variables, are events or constructs that change during therapy and whose change can lead to subsequent changes in outcome. Focusing on trait-like components as baseline for therapy and state-like components that are needed to influence the course of treatment can lead to personalised treatment recommendations. Knowing how treatment works and what mechanisms of change work for whom, can improve our treatments and eliminate ingredients of treatment that do not work (Zilcha-Mano, 2021). Therefore, finding out if there are also these mechanisms of change in play for treatment failure can also enhance the personalisation of therapy.

This scoping review provides an overview of the outcomes of studies on predictors of treatment failure (defined as treatment failure, deterioration, non-response, or drop-out for psychotherapy) in the treatment of adult patients with anxiety and depressive disorders. We chose to use this broad definition of treatment failure to capture all relevant studies addressing this topic as comprehensively as possible. Narrowing the definition too strictly carries the risk of excluding well-conducted research that could contribute valuable insights into the predictors of poor treatment outcomes. Next to that, we specifically focused on predictors that are patient-related and patient-specific, as such features are expected to be more amenable to change before starting therapy than demographic or disorder-specific features, and (thus) may be useful targets to reduce treatment failure.

## Method

### Literature Search

In order to review the available body of knowledge concerning patient-specific predictors for treatment failure, a literature search in PubMed, PsycInfo (OVID interface, 1806 onwards), Embase (OVID interface, 1974 onwards), and Medline, and Pre-Medline (OVID interface, 1946 onwards) was conducted with help from a research librarian. This search was aimed at articles that combined the following search criteria and terms using subject headings (MeSH for Medline, Emtree for Embase, and APA thesaurus for PsycInfo), if available: predictors, treatment failure, depressive or anxiety disorders, and psychotherapy. Treatment failure was further defined as poor outcome, non-response, drop-out, deterioration, or not successful as the search criteria. The literature search was confined to English, German, and Dutch. A draft of the search strategy in Embase is included in Table 1.

**Table 1:** Draft Search in Embase on November 14, 2017

#^a^	Keywords used in the search	Results^b^
1	exp *anxiety disorder/	93842
2	*anxiety/	48269
3	1 or 2	137512
4	*depression/ or *agitated depression/ or *atypical depression/ or *dysphoria/ or *dysthymia/ or *endogenous depression/ or *involutional depression/ or *late life depression/ or *major depression/ or *masked depression/ or *melancholia/ or *"mixed anxiety and depression"/ or *organic depression/ or *perinatal depression/ or *postoperative depression/ or *premenstrual dysphoric disorder/ or *puerperal depression/ or *reactive depression/ or *recurrent brief depression/ or *seasonal affective disorder/ or *treatment resistant depression/	167039
5	3 or 4	281358
6	treatment failure/	91108
7	("poor outcome" or failure or failing or deteriorat* or worse* or unsucces* or "nonsucces*" "not succesful" or "no success" or drop-out* or drop out* or regress* or quit* or non-respons* or "non repsons*").mp.	2684336
8	deterioration/	34929
9	6 or 7 or 8	2684336
10	5 and 9	36039
11	prediction/ or "prediction and forecasting"/ or adverse outcome/ or forecasting/ or prediction/ or predictive validity/ or predictive value/ or prognosis/	1004236
12	10 and 11	3518
13	exp *psychotherapy/	116310
14	12 and 13	144
15	limit 14 to (dutch or english or german)	143

*Note.* Search words ending with / used the thesaurus (subject headings) of Embase, using "" forces the search to use words like “and” or “no” as a search term instead of a command. * Before the word is a focus command, which indicates that the search focuses on articles where the search word is the main topic. *Behind the word is a truncation command and indicates that it searches for variations on a word with different suffixes. Exp = explodes, meaning that it expands the search results of terms entered and includes more specific related topics. .mp = multiple fields, indicating these search terms are searched only in the most useful fields (e.g., title, abstract, keywords).

^a^number referring to a single search. ^b^number of articles found with this specific search term on that specific date.

The last search was conducted on October 2, 2025, earlier searches were conducted in March 2022 and November 2017. We included studies examining a form of psychotherapy among adult populations (older than 18 years) with anxiety and depressive disorders.

### Selection Process

**Figure 1:**
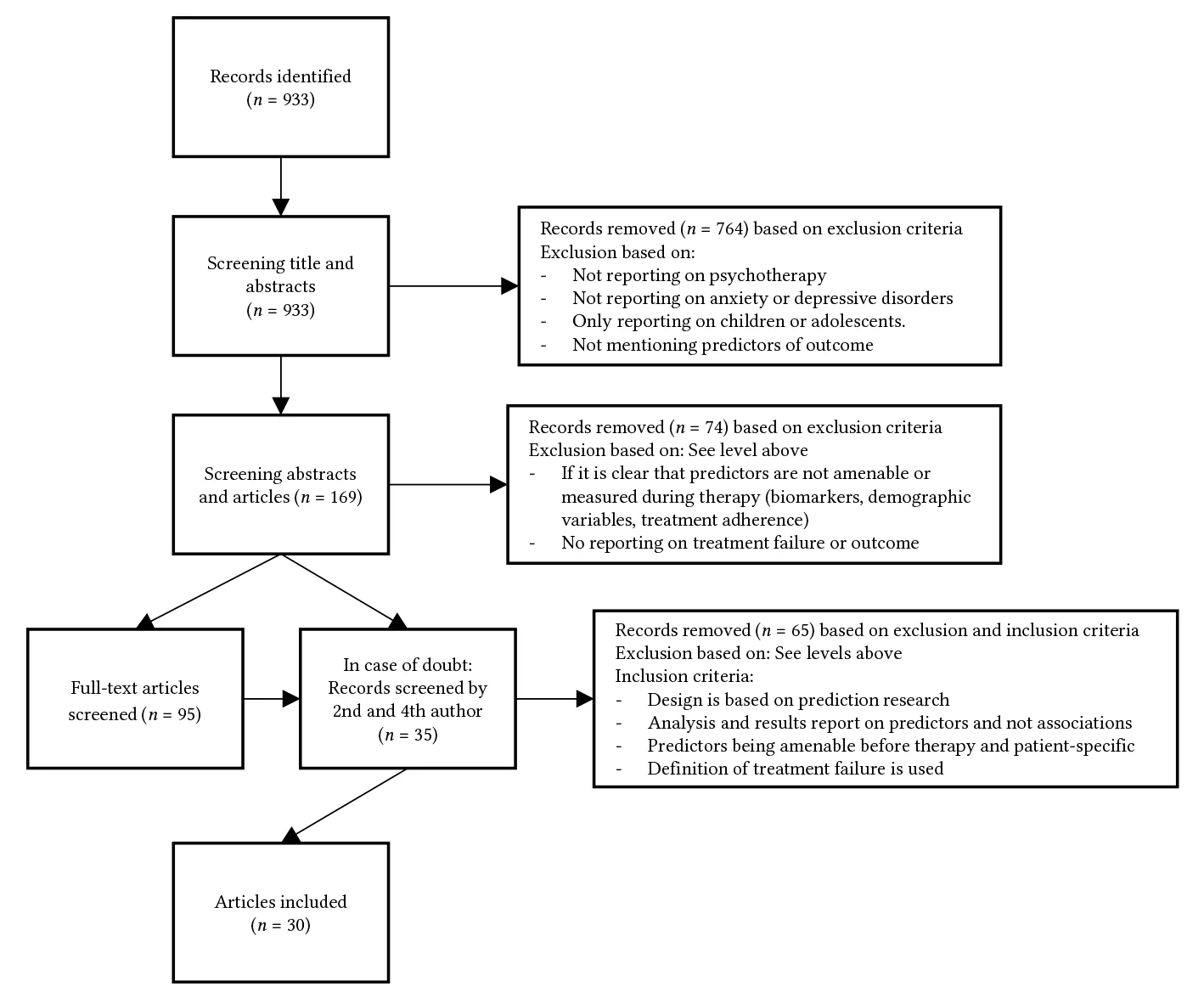
Prisma Flow Chart

We found 933 articles within this search. Figure 1 shows the PRISMA flow chart. Out of all these articles, the first author (VP) made a further selection. First, all titles and abstracts were checked for exclusion criteria. They were excluded if there was no mentioning of psychotherapy, anxiety, or depressive disorders, or prediction of outcome. They were also excluded if they only reported on child and adolescent studies.

Next, the remaining 169 articles were screened by reading the abstracts and the conclusions for meeting exclusion criteria. Articles were excluded based on the above-described exclusion criteria, and when predictors were not amenable or were measured during therapy and not at the start of therapy. Articles were also excluded when there was no mentioning of treatment failure or outcome. The remaining 95 papers were inspected more precisely by reading them fully, in order to check for reported amenable predictors of treatment failure. If it was not clear what kind of predictors were used and how they were measured, the methods and results sections were read carefully to inspect the analyses in order to find out if the variables could be labeled as predictors. Articles were included when they reported on treatment failure, predictors being amenable before therapy, and patient-specific, and when studies were based on prediction analyses. To decrease the risk of bias, in case of doubt, the second and fourth co-author also read the articles before including or excluding them. This was the case in 35 articles. In total, 30 papers met the inclusion criteria and were included in this review. Table 2 summarizes these articles.

**Table 2:** Overview of Included Articles

Article	Measurement of treatment failure	Sample size^a^	Treatment	Target group^b^	Predictors and measurement
Arndt et al. (2020)	Drop-out: Termination of treatment before week 12 of the intervention	Total: 1013 Intervention: 509 Control: 504	12-week internet-based CBT intervention and Control group	Adults (18-65) with depressive symptoms	Treatment Attitude (Attitudes Towards Psychological Online Interventions Questionnaire), Physical health (Short-Form Health Survey)
Bélanger et al. (2017)	Drop-out: discontinuation of therapy. Difference made between drop-out before treatment, in the first 7 sessions and after 8 sessions	Total: 77	CBT	Adults (18-65) Panic disorder with agoraphobia	Treatment expectations (Process Expectations Questionnaire), Dyadic adjustment (Dyadic Adjustment Scale)
Blom et al. (2007)	Drop-out. (Outcome: residual gain & norm score)	Total: 193	12 sessions IPT, medication and minimal contact, 12 sessions IPT and medication, 12 sessions IPT and pill-placebo	Age >18 Non-psychotic, non-bipolar major depressive disorder	Personality traits (NEO-Five Factor Inventory)
Chambless & Steketee (1999)	Drop-out: Leaving treatment before receiving at least 10 sessions of treatment	Total: 101	22 sessions in 16 weeks	Adults (18-65) with obsessive compulsive disorder or panic disorder with agoraphobia	Perceived criticism (Perceived Criticism Measure), hostility (Camberwell Family Interview), expressed emotion (Relatives Reactions Questionnaire, Composite Measure of Emotional Overinvolvement)
Chambless et al. (1997)	Residual gain: change with treatment adjusted for pretreatment severity levels	Total: 64	Group CBT	Social Phobia	Treatment expectancy (Treatment Expectancy Scale)
Critchfield et al. (2007)	End state functioning: normal range score on zero to three of the six measures	Total: 24 Cognitive therapy: 10 Applied relaxation: 6 Combination: 8	14 sessions CBT, three different variants of CBT: cognitive therapy, applied relaxation, and combination	Generalized anxiety disorder	Interpersonal process between therapist and client. (Structural Analysis of Social Behavior coding system)
Grilo et al. (1998)	Drop-out: not completing the 11-session therapy	Total: 162	Individual 11- session CBT, pharmacotherapy or combined treatment	Panic disorder uncomplicated or with mild agoraphobia	Illness/treatment attributions (Treatment Attitude Measure; Etiological Model Questionnaire) Coping styles (Ways of Coping Checklist) Personality styles (Wisconsin Personality Inventory)
Hoyer et al. (2016)	Non-response: Less than 31% reduction of anxiety symptoms Drop-out: all patients who stopped treatment or assessments	Total: 244	Up to 30 individual sessions cognitive therapy	Age: 18-70 Primary diagnosis social anxiety disorder	Personality dimensions (Tri-Dimensional Personality Questionnaire) Self-esteem (subscale of the Frankfurt Self Concept Scales) Shame (subscale of Test of Self-conscious Affects) Interpersonal problems and attachment style (Inventory of Interpersonal Problems)
Johnson et al. (2014)	Drop-out: Missing more than one or two sessions (depending on the treatment)	Total: 74 Virtual reality exposure: 32 Exposure group therapy: 42	8 sessions virtual reality exposure therapy or exposure group therapy	Social anxiety disorder with a primary fear of public speaking	Stereotype confirmation concerns (Stereotype Confirmation Concerns Scale)
Keefe et al. (2021)	Drop-out: not completing at least 16 sessions in 12 weeks	Total: 200 Psychodynamic therapy: 80 CBT: 80 Applied relaxation therapy: 40	CBT & Psychodynamic therapy & applied relaxation training total of 24 sessions in 12 weeks	Adults (18-70) Panic disorder with or without agoraphobia	Treatment expectancies (Expectancy Scale)
Keijsers et al. (1994)	Treatment failure: improvement equal or less than 30%	Total: 51	18 sessions exposure in vivo and exposure with response prevention	Obsessive compulsive disorder	Motivation for treatment (Willingness to Participate Scale of the Nijmegen Motivation List)
LeBeau et al. (2013)	Improvement of clinical severity ratings	Total: 84 CBT: 48 Acceptance and commitment therapy: 36	12 sessions CBT or acceptance and commitment therapy	Anxiety disorder Age 18-60	Treatment expectations (modified form of The Credibility/Expectancy Questionnaire)
Lin & Farber (2021)	Latent growth mixture modelling Treatment Outcome Package depression score	Total: 63	Different psychotherapies for over 9 months (i.e. psychodynamic, CBT, dialectical behavior therapy)	Depression	Self-concealment (Self-Concealment Scale)
Lutz et al. (2019)	Drop-out: non-consensual and non-recommended termination of therapy	Total: 1,234	Average 30.85 sessions individual psychotherapy	Age: 14-76 Different DSM-IV diagnoses with SCID-I	Personality style (Persönlichkeits-Stil- und Störungs-Inventar (PSSI-K)) Treatment expectations by patient and therapist (one-item question) Interpersonal problems (Outcome Questionnaire 30, Questionnaire for the Evaluation of Psychotherapeutic Progress)
Marker et al. (2019)	Drop-out: if session 10, 11 and 12 were not attended	Total: 58	12 sessions transdiagnostic Group-CBT for anxiety	Anxiety disorders Age: 19-58	Readiness to change (University of Rhode Island Change Assessment Scale) Motivation/change talk (Client Language Easy Rating Coding System)
Marquett et al. (2013)	Significant clinical improvement: no diagnosis (MINI) or non-depressed range on the questionnaires	Total: 60	12 sessions individual CBT over 3-4 months	Age: 60 and above Major or minor depression and dysthymic disorder	Impact of stressful events (Elders Life Stress Inventory, Impact of Events Scale -6, The Integration of Stressful Life Event Scales), Social support (Abbreviated Duke Social Support Index), Locus of control (Rotter’s Locus of Control theory), Personality (Big Five Inventory), Coping style (The Brief Cope)
Miller et al. (1996)	Non-response: score of 11 or more on the Hamilton Depression Rating Scale after 26 weeks of treatment	Total: 61	Up to 26 weeks of treatment, medication, IPT, or a combination	Elderly with recurrent depressive disorder	Perception of illness. (Perception of Illness Scale)
Moggia et al. (2020)	Growth mixture modelling (with Clinical Outcome in Routine Evaluation—Short Form B & Beck Depression Inventory-II)	Total: 108	7 sessions CBT group therapy and after that Individual CBT or Individual dilemma focused therapy	Age 18-70, Major depressive disorder or dysthymic disorder	Self-ideal discrepancy (Repertory Grid Technique: system to interpret the self, others and the world)
Moradveisi et al. (2014)	Drop-out: not completing therapy	Total: 100 Behavioral activation: 50 Anti-depressant medication: 50	16 sessions in 12 weeks behavioral activation or 12 weekly sessions anti-depressant medication	Major depressive disorder Age 18-60	Preference/attitudes towards therapy (Preference-Attitude Questionnaire)
Parker et al. (1986)	Improvement of depression	Total: 91 Used for predictive value of coping questionnaire: 48	Psychiatric consults	Depressive disorder	Coping behavior (Coping Questionnaire)
Renaud et al. (2014)	Change in clinical global impression score	Total: 256	Average of 19 CBT sessions	Anxiety or depressive disorder	Treatment expectancy (Suitability for Short-Term Cognitive Therapy)
Safren et al. (1997)	Improvement after treatment Drop-out	Total: 113	Group CBT	Social phobia	Expectations of treatment (Reaction to Treatment Questionnaire)
Schilling et al. (2021)	Not on track: Failure boundary per session, based on Hopkins-Symptom-Checklist-11 scores	Total: 413 Control group: 157 Feedback group: 256	CBT	Adults with different diagnoses	Motivation (Assessment for Signal Clients), social support (Assessment for Signal Clients), emotion regulation (Affective Style Questionnaire)
Schindler et al. (2013)	RCI & quality associated drop-out, not completed number of allowed sessions	Total: 193	CBT	Major depressive disorder or dysthymic disorder	Treatment expectancies (one-item question)
Solomonov et al. (2021)	Latent Growth Mixture Models Early non-response: minimal change in depression severity	Total: 221 Problem-solving therapy: 107 Supportive therapy: 111	12 weeks problem-solving therapy or supportive therapy	Adults >60 years Non-psychotic major depression disorder	Neuroticism (NEO-Personality Inventory) Treatment expectancy (4-item Treatment Rationale Scale) Perceived social support (four subscales of the Duke Social Support Index)
Steketee et al. (2011)	Drop-out: completed less than 18 sessions	Total: 39 Uncontrolled pilot trail: 10 Waitlist controlled trial: 29	22 sessions cognitive therapy	Obsessive compulsive disorder	Personality traits (Personality Diagnostic Questionnaire-4) Motivation (University of Rhode Island Change Assessment Scale) Treatment expectancy (Expectancy Rating)
Strauss et al. (2017)	Drop-out: premature treatment discontinuation	Total: 412 CBT: 213 Psychodynamic therapy: 199	25 individual sessions psychodynamic therapy 30 sessions CBT	Age 18-70 Primary diagnosis: social anxiety disorder	Experiences in close relationships (Experiences in Close Relationships-Revised Questionnaire)
Vogel et al. (2006)	Improvement between pre- and posttreatment measures	Total: 37	Twice weekly individual exposure and response prevention sessions for 6 weeks	Obsessive compulsive disorder with overt compulsions	Treatment expectancy (one-item question) Motivation to change (The University of Rhode Island Change Assessment Scale)
Westra (2011)	Improvement between pre- and posttreatment measures on Penn State Worry Questionnaire	Total: 38 Motivational Interviewing: 19 No Motivational interviewing: 18	14 hours CBT group 6 weekly 2-hour sessions followed by 2 1-hour sessions Motivational Interviewing pretreatment condition: 4 individual weekly sessions	Generalized anxiety disorder Age 18-66	Motivation (Change Questionnaire), Motivation to change (Client Motivation for Psychotherapy Scale), Resistance to therapy (Client Resistant Code)
Zilcha-Mano et al. (2016)	Drop-out: failure to complete the 16-week treatment protocol	Total: 156 Short-term Psychodynamic therapy: 51 Medication: 55 Placebo: 50	16 weeks 20 sessions short-term psychodynamic therapy, medication (sertraline) or placebo.	Major depressive disorder	Working alliance expectations (Working alliance inventory), Interpersonal Problems (Inventory of Interpersonal Problems-Circumplex)

^a^Sample size is total number of included patients, some articles specify these in different subsamples. ^b^Age is always 18 plus, if not otherwise described.

### Primary Outcomes

The main outcome of this review will be the predictors of treatment failure. A predictor was included if the variable was amenable to change and patient-specific, such as motivation or treatment attitude. As such, biological markers, demographic variables, comorbidity, or severity of complaints were excluded. We included only predictors of treatment failure, not predictors of follow-up or risk of relapse.

To improve readability, we categorized the predictors into categories based on both their definition and the instrument used to measure them in the studies included in the review (e.g., Motivation measured with the URICA). For instance, seeking social or emotional support, as mentioned by Grilo et al. (1998) and Marquett et al. (2013), was defined as a coping style and measured with a coping questionnaire. In contrast, perceived social support, mentioned by Marquett et al. (2013), Schilling et al. (2021), and Solomonov et al. (2021), was measured with instruments focusing on how patients perceive social support. This construct was therefore considered distinct from coping. Because of this conceptual distinction, we placed these seemingly similar constructs in different categories to ensure that each predictor reflected the specific theoretical framework and measurement approach used in the studies. Through this process, we specified seven predictor categories and one category “other” for predictors that did not fit elsewhere. The identified predictor categories are personality factors, coping strategies, motivational aspects, attributions, treatment attitude and expectancies, interpersonal relationships, and others.

## Results

An important problem in the literature on treatment failure is the lack of consensus on its definition, which makes comparisons of studies difficult. This problem is reflected in the studies included in this review. Eleven of the included articles (37%) defined treatment failure as insignificant improvement in complaints, four of them used an additional component to define non-response, such as norm scores or a decrease percentage. Four studies (13%) defined treatment failure as residual gain, i.e., the individual difference in improvement of complaints controlled for the expected difference based on the pre-test score, indicating that treatment failure is viewed as a continuous variable in these studies. Two of these studies used an additional component to define treatment failure, such as norm scores or a decrease in percentage. Another three studies (10%) used norm scores of their primary outcome measure to determine treatment failure, one study (3%) used the reliable change index as formulated by Jacobson and Truax (1991) to define treatment failure, whereas one study (3%) stated that treatment failure was less than 50% decrease in complaints. In the remaining ten of the studies (34%), treatment failure was defined as drop-out from treatment.

In this results section, we will describe the findings of all studies per predictor category. In Table 3, we specify for each predictor, the papers that report them as predictors of treatment failure.

**Table 3:** Summary of Predictors of Treatment Failure

Predictor	Deterioration	Drop-Out	Non-Response
Personality traits			
Histrionic personality style		+ Lutz et al. (2019)	
Neuroticism		+ Blom et al. (2007)	+ Solomonov et al. (2021)
Openness to experiences			- Marquett et al. (2013)
Obsessive personality style		- Lutz et al. (2019)	
Coping strategies			
Use and seeking social or emotional support		- Grilo et al. (1998)	- Marquett et al. (2013)
Self-consolation			+ Parker et al. (1986)
Motivation			
Motivation	- Schilling et al. (2021)		- Westra (2011) - Keijsers et al. (1994)
Resistance to therapy			+ Westra (2011)
Change talk		- Marker et al. (2019)	
Attributions			
To life stressors		+ Grilo et al. (1998)	
Treatment attitude and expectancies			
Treatment attitudes		- Grilo et al. (1998) - Arndt et al. (2020)	
Outcome expectancy		- Schindler et al. (2013) - Solomonov et al. (2021) - Keefe et al. (2021)	
Expectations of a strong alliance		- Zilcha-Mano et al. (2016)	
Process expectations		+ Bélanger et al. (2017)	
Interpersonal relationships			
Emotional overinvolvement and hostility		+ Chambless & Steketee (1999)	
Impairment of interpersonal relationships		+ Lutz et al. (2019)	
Perceived social support			- Solomonov et al. (2021)
Vindictive tendencies			+ Zilcha-Mano et al. (2016)
Perceptions of acceptation by the therapist			- Solomonov et al. (2021)
Other			
Negative impact of stressful events			+ Marquett et al. (2013)
External locus of control			+ Marquett et al. (2013)
Assign blame for stressful events to others			- Marquett et al. (2013)
Physical health		- Arndt et al. (2020)	
Stereotype confirmation concerns		+ Johnson et al. (2014)	

*Note.* - = low score on the predictor, + = high score on the predictor.

### Personality Factors

Six studies examined personality factors in relation to treatment failure. Three of them used large samples of patients with depressive disorders (Blom et al., 2007; Marquett et al., 2013; Solomonov et al., 2021). Solomonov et al. (2021) studied the Big Five personality traits in problem-solving therapy and supportive therapy for late-life depression (age > 60) with executive dysfunction. Blom et al. (2007) used a sample of depressive adults following interpersonal psychotherapy. Both Blom et al. (2007) and Solomonov et al. (2021) found that only higher scores on neuroticism are a predictor for treatment failure. The other personality traits of the Big Five (i.e., extraversion, openness to experiences, conscientiousness, and agreeableness) were not found to be predictive of treatment failure (defined as drop-out and early non-response; Solomonov et al., 2021). Marquett et al. (2013) found that a lower score on openness to experience is a predictor of treatment failure (no significant clinical improvement) in a small sample of late-life depression (age > 60) treated with CBT.

Three studies focused on personality styles or indicators instead of traits (Grilo et al., 1998; Hoyer et al., 2016; Lutz et al., 2019). Only a more histrionic personality style and a less obsessive personality style predicted a higher probability of treatment failure (drop-out) in CBT for several diagnoses, with mostly affective, personality, and anxiety disorders (Lutz et al., 2019). Other predictors like harm-avoidance, self-esteem, shame, novelty seeking, dominance, and reward dependence were not found as predictors of treatment failure (treatment response and drop-out) in cognitive therapy for social phobia (Hoyer et al., 2016). Neither were personality styles, self-reported features from personality disorders from an internal perspective, such as avoidant or narcissistic personality styles, predictors of treatment failure in cognitive behavioral therapy, medication therapy, or combination treatment for panic disorder (Grilo et al., 1998).

### Coping Strategies

Several coping strategies were studied as predictors of outcome. It is remarkable that all studies used different instruments to measure coping styles or strategies.

The use of seeking social or emotional support is the only coping strategy that was found to be predictive of treatment failure (drop-out and no clinically significant improvement) in more than one study (Grilo et al., 1998; Marquett et al., 2013). Samples used in these studies were a small sample of depressive older adults (age >60) following CBT (Marquett et al., 2013) and patients with uncomplicated panic disorder or with mild agoraphobia following CBT, pharmacotherapy, or combined treatment.

Other coping strategies were only studied as a predictor in one article and in different patient samples. Of those strategies, only higher self-consolation (with behavior like spending money, eating more, and drinking more alcohol) was associated with treatment failure (a high likelihood of not improving) after psychotherapy in depressed patients (Parker et al., 1986). Other coping strategies turned out to be not predictive of treatment failure. The Grilo et al. (1998) study revealed that confrontive coping, distancing, self-control, acceptance of responsibility, escape avoidance, problem solving, positive reappraisal, problem focusing, self-blame, wishful thinking and avoidance were not associated with treatment failure (drop-out) in the treatment of patients with a panic disorder. Finally, Lin and Farber (2021) found that self-concealment (the tendency to conceal distressing information aiming at self-protection and avoidance of stigma) measured at the baseline of psychotherapy, was not predictive of treatment failure (non-improvement) in depressive complaints.

### Motivational Aspects

Findings about motivation as a predictor of treatment failure are contradictory. For instance, Keijsers et al. (1994) found that poor motivation predicted treatment failure (no improvement) in obsessive compulsive disorder (OCD) treatment. In line with these findings Schilling et al. (2021) found that a sudden drop in therapy motivation was predictive for treatment failure (deterioration of complaints) in CBT for different diagnosis, although therapy motivation did not differ between on-track and not on-track patients. However, motivation was found not to be predictive of treatment failure (no improvement) of CBT worry treatment (Westra, 2011). All three studies used different questionnaires for measuring motivation.

Readiness for change, measured with the URICA, is seen as a different conceptualization of motivation. However, readiness for change was not predictive of treatment failure (no improvement and drop-out) in small samples of CBT for OCD and anxiety disorders (Marker et al., 2019; Steketee et al., 2011; Vogel et al., 2006).

Finally, change talk and counter change talk i.e., patient statements indicating support or opposition to change, an observational measure of motivation (Marker et al., 2019), did not predict treatment failure (drop-out) during early sessions of therapy in which psychoeducation and cognitive restructuring were offered. However, low change talk did predict treatment failure (drop-out) during exposure sessions later in CBT for anxiety disorders. Next to that, Westra (2011) found that low motivation to change and higher resistance to therapy, considered by Westra (2011) to be an indirect measure of motivation, were predictive of treatment failure (no improvement) in a small GAD-sample treated with CBT.

### Attributions

There are only two studies on attributions as predictor of treatment failure. They both study a different sort of attribution. Attributing their panic disorder to life stressors predicted treatment failure (drop-out) for patients receiving treatment for panic disorder (Grilo et al., 1998). Perception of health, defined as the way people rate their overall physical health, did not predict treatment failure (non-response) in a group of late-life depressive patients who received IPT (Miller et al., 1996).

### Treatment Attitude and Expectancies

Several studies found that treatment expectancy did not predict treatment failure (Chambless et al., 1997; LeBeau et al., 2013; Lutz et al., 2019; Renaud et al., 2014; Safren et al., 1997; Steketee et al., 2011). These outcomes are replicated in several studies with big sample sizes and different diagnosis.

On the other hand, outcome expectancy is found as a predictor of treatment failure in multiple studies (Keefe et al., 2021; Schindler et al., 2013; Solomonov et al., 2021). Patients with a more negative outcome expectancy were found to be more likely to have treatment failure (drop-out) for depression (Schindler et al., 2013; Solomonov et al., 2021). In a study comparing three treatments for panic disorder (CBT, focused psychodynamic therapy and applied relaxation), Keefe et al. (2021) found that outcome expectancies measured at session two were not predictive of treatment failure (drop-out) in the overall study. However, they did find that in the CBT group, lower outcome expectancies at session two were predictive of treatment failure (drop-out), which was not found for the other treatment conditions. Contrary to these findings, Vogel et al. (2006) did not find outcome expectancy as a predictor of treatment failure in a small sample of OCD patients with overt compulsions.

There are also predictors related to treatment expectancies that were only found in one study but not replicated in other studies. Patients with lower expectations of a strong alliance had a higher risk at treatment failure (drop-out) during supportive-expressive therapy than during medication or placebo therapy (Zilcha-Mano et al., 2016). In the early phase of panic disorder treatment, in which psychoeducation and cognitive restructuring were offered, higher process expectations (i.e., expectations on the therapy process and role expectations) were predictive of treatment failure (drop-out), but anxiety expectations (i.e., expectations on having panic-related symptoms) were not. In the behavioral phase of treatment, where exposure and anxiety-provoking exercises took place, anxiety and process expectations were not predictive of treatment failure (drop-out) (Bélanger et al., 2017).

Treatment attitude implies the way people think of certain therapies or views they have on psychotherapy in general. Treatment attitude is studied in depressive and panic disorder patient populations with large sample sizes (Arndt et al., 2020; Grilo et al., 1998; Moradveisi et al., 2014). Moradveisi et al. (2014) found that treatment attitudes are no predictor of treatment failure. Contrary to that, treatment failure (attrition, or the likelihood of drop-out) could be predicted by less favorable or negative treatment attitudes towards the offered treatment, found in an internet-based intervention for depression and panic disorder treatment (Arndt et al., 2020; Grilo et al., 1998).

### Interpersonal Relationships

Interpersonal relationships are a broad concept and include for example social support and attachment characteristics. Several studies found aspects of interpersonal relationships to be predictive of treatment failure. The only predictor within this category studied in multiple studies is perceived social support (Marquett et al., 2013; Schilling et al., 2021; Solomonov et al., 2021). Whereas Schilling et al. (2021) and Marquett et al. (2013) did not find perceived social support as a predictor of treatment failure in depression and other diagnosis, Solomonov et al. (2021) found low perceived social support to be predictive of treatment failure (early non-response) in psychotherapy for late-life depression.

Other interpersonal factors that were found as predictors for treatment failure are emotional overinvolvement from relatives and hostility coming from relatives (Chambless & Steketee, 1999) as well as higher impairment of interpersonal relationships (Lutz et al., 2019). Both were found to predict higher rates of drop-out in CBT for several diagnosis. Higher vindictive tendencies in interpersonal relationships were found to be predictive of treatment failure (drop-out) in pharmacotherapy for depressive disorder, but not in other treatment groups (Zilcha-Mano et al., 2016). Lastly, Solomonov et al. (2021) found that perceptions of the therapist as less accepting are predictive of treatment failure (early non-response) in psychotherapy for late-life depression.

However, not all studies found interpersonal relationships to be predictive of treatment failure. Although low end state functioning groups had higher levels of interpersonal hostility, Critchfield et al. (2007) found no predictors in interpersonal process behaviors in a small sample of patients with generalized anxiety disorder (GAD) receiving CBT. Further, dyadic adjustment, which focuses on consensus, satisfaction, cohesion, and expression of affection within a romantic relationship, did not predict treatment failure (dropout) in CBT for panic disorder (Bélanger et al., 2017). Perceived criticism was no predictor of treatment failure in treatment for patients with OCD or panic disorder (Chambless & Steketee, 1999). Hoyer et al. (2016) did not find interpersonal problems and attachment style as predictors of treatment failure in cognitive therapy for patients with social anxiety disorder. Finally, Strauss et al. (2017) found that partner-related attachment anxiety and avoidance in romantic relationships are no predictors of treatment failure (drop-out) in both CBT and psychodynamic therapy for patients with social anxiety disorder.

### Other Categories

Several predictors that were studied in the included papers could not be classified under one of the categories of predictors discussed so far. Notably, none of those predictors were studied in multiple studies, whereas a number of the target groups in these studies were very specific, for example late life depression, or social anxiety disorder with a primary fear of public speaking.

First of all, patients who experience a high negative impact of stressful events are more likely to experience treatment failure (non-response) to CBT for late-life depression (Marquett et al., 2013). The same was true for an external locus of control. Interestingly, also patients who do *not* tend to assign blame for stressful events to others (decreased external blame) turned out to be more likely to experience treatment failure (non-response) (Marquett et al., 2013). Further, Arndt et al. (2020) studied different drop-out patterns in an internet-based depression intervention and found that low physical health increases the risk to belong to the drop-out group, whereas Johnson et al. (2014) found that stereotype confirmation concerns, defined as being afraid of confirming certain stereotypes leading to negative evaluations, are associated with higher risk of treatment failure (drop-out) in social anxiety disorder treatment. Moggia et al. (2020) measured self-ideal discrepancy, which is the difference between the rating of the ideal self and the self as it is now, self-others discrepancy, explained as how someone views him/herself in respect to others, and a dilemmatic construction of the self, which means that a person desires a change in one construct of the self that correlates with an undesirable change in another construct of the self. All of these measures appeared not to be predictive of treatment failure (non-improvement) in psychotherapy for depression. Finally, Schilling et al. (2021) found that emotion regulation was no predictor of treatment failure (deterioration of complaints) after CBT for different diagnosis.

## Discussion

### Summary of Results

In this paper, we reviewed studies on predictors of treatment failure (also defined as non-response, deterioration, and drop-out) for depressive and anxiety disorders. We specifically focused on amenable patient-specific predictors, such as motivation or coping styles. Knowledge of such factors might help us reduce treatment failure and thus improve treatment outcomes. Like Steketee and Chambless (1992) and Eilertsen and Eilertsen (2023), we encountered several problems within the existing literature. One important problem is the fact that both treatment failure and predictors are measured differently, which makes studies difficult to compare. Another problem is a lack of replication studies, and if predictors were replicated, this was done in studies using small or specific patient samples (e.g., elderly, social phobia with fear of speaking in public, or OCS with overt compulsions). Consequently, it is difficult to conclude the overall impact of these predictors.

However, a few predictors that stand out are treatment expectancy (replicated in three studies with a big sample size and different patient samples), neuroticism (replicated in two studies with the same instrument in different patient samples), the use of social support (replicated in two studies with different measuring instruments), readiness to change (replicated in three studies, in very small patient samples), outcome expectancy (two studies with different measuring instruments), and perceived social support (three studies, but only in late-life depression found as a predictor).

Focusing more on the different definitions of treatment failure, we see that about half of the included articles focus on drop-out and the other half on non-response. There are two articles that mention deterioration. Because drop-out, non-response, and deterioration are different constructs, we also looked at the difference in predictors per treatment failure definition. We see that almost none of the predictors are replicated within these treatment failure categories. The only predictors that remain are readiness to change and treatment expectancy. Readiness to change was studied in three different studies, two of which focused on drop-out, and both studies found that it was no predictor of drop-out. Treatment expectancy was studied in six different studies, all of which found that it was not a predictor of drop-out (three studies) or non-response (three studies). However, drop-out and non-response in all these studies were measured differently, which still makes it hard to draw firm conclusions about the predictive validity of these measures.

### Clinical Implications

Targeting the identified predictors before or early in the treatment of anxiety and depressive disorders might help reduce treatment failure, which in turn might increase the cost-effectiveness of treatments. For example, in accordance with Constantino et al. (2018), assessing the outcome expectancy of patients at the start of treatment gives insight into the probability of a poor treatment outcome or even treatment failure. It offers an opportunity to assess whether interventions targeting the expectations of treatment should be integrated in the treatment in order to reduce the risk of treatment failure. Also, by assessing the use of social support early in treatment, interventions can be focused more on involving the social support system in therapy or enhancing coping strategies for asking for help, which might prevent treatment failure.

Unfortunately, as there are very few to no amenable factors that show strong evidence of being a predictor of treatment failure, clinicians need to be careful to use variables of which they assume they influence a poor therapy outcome. With the current knowledge, it is not possible to withhold patients from specific treatments or adapt treatments based on amenable predictors of treatment failure.

### Research Implications and Suggestions

One important limitation is that for each predictor, only a limited number of studies have been carried out, often for only one specific treatment and/or disorder. For instance, CBT was the treatment of choice in five of the six studies into treatment expectancy as a predictor, whereas only two of them studied a sample of patients with the same disorder. As we cannot rule out the possibility that predictors of treatment failure differ for different disorders as well as for different treatments, predictors preferably should be studied in multiple treatment and patient groups to reach more definitive conclusions on overall predictors of treatment failure (Dalgleish et al., 2020). A further limitation is the absence of standardized measures for response rates (Loerinc et al., 2015), which makes it difficult to draw definitive conclusions about treatment outcome and -thus- about treatment failure. The reliable change index (RCI), as defined by Jacobson and Truax (1991), could address this problem (Loerinc et al., 2015), as by using this index treatment failure can be defined as meeting the criteria for no clinically significant change or deterioration at the end of treatment. The fact that the vast majority of studies reporting on predictors of treatment outcome only studied (or reported) predictors of treatment success or reported on poor(er) outcome is a third limitation. As we could not assume that poor(er) outcome is identical to treatment failure, or that results of treatment success can be inverted to treatment failure, most studies had to be excluded. Future studies would benefit from also studying and reporting on predictors of treatment failure instead of treatment success only. Another limitation is that the terms ‘association’ and ‘prediction’ were used interchangeably in both the results and discussion sections of papers excluded from this review. Hence, it was not always clear if the studied variable was in fact a predictor, or if the predictors were just correlated with the outcome. We only included the studies that were very clear that they measured predictors of treatment failure. The field would benefit from a more consistent and precise use of these terms in presenting results on predictors of treatment outcome. A final limitation is that this review is not pre-registered and is not completely written according to current scoping review guidelines (Tricco et al., 2018). This stems from the fact that the first search of this review was conducted in 2017, before the publication of these guidelines. We recommend a future scoping or systematic review on the same topic to replicate and renew these findings.

### Conclusion

By knowing which factors are predictors of treatment failure, we can move towards studying how to intervene on these factors in order to decrease the odds of treatment failure. Based on the results of this review, there are no predictors that stand out as overall strong amenable predictors of treatment failure. Future studies are needed to determine whether already found predictors are replicable with the same instruments in different patient samples and with different treatments.

## Data Availability

Data sharing is not applicable to this article as no new data were created or analysed in this study.

## Reporting Guidelines

The Prisma ScR guidelines were followed in writing this scoping review (Tricco et al., 2018).

## Preregistration

There is no preregistration of this manuscript.

## Social Media Accounts

*Vivian Peerbooms:* LinkedIn
